# Sugarcane stem node identification algorithm based on improved YOLOv5

**DOI:** 10.1371/journal.pone.0295565

**Published:** 2023-12-11

**Authors:** Zhongjian Xie, Yuanhang Li, Yao Xiao, Yinzhou Diao, Hengyu Liao, Yaya Zhang, Xinwei Chen, Weilin Wu, Chunming Wen, Shangping Li

**Affiliations:** 1 College of Electronic Information, Guangxi Minzu University, Nanning, China; 2 Guangxi Key Laboratory of Hybrid Computation and IC Design Analysis, Guangxi Minzu University, Nanning, China; 3 Guangxi Key Laboratory of Machine Vision and Intelligent Control, Wuzhou University, Wuzhou, China; Khon Kaen University, THAILAND

## Abstract

Identification of sugarcane stem nodes is generally dependent on high-performance recognition equipment in sugarcane seed pre-cutting machines and inefficient. Accordingly, this study proposes a novel lightweight architecture for the detection of sugarcane stem nodes based on the YOLOv5 framework, named G-YOLOv5s-SS. Firstly, the study removes the CBS and C3 structures at the end of the backbone network to fully utilize shallow-level feature information. This enhances the detection performance of sugarcane stem nodes. Simultaneously, it eliminates the 32 times down-sampled branches in the neck structure and the 20x20 detection heads at the prediction end, reducing model complexity. Secondly, a Ghost lightweight module is introduced to replace the conventional convolution module in the BottleNeck structure, further reducing the model’s complexity. Finally, the study incorporates the SimAM attention mechanism to enhance the extraction of sugarcane stem node features without introducing additional parameters. This improvement aims to enhance recognition accuracy, compensating for any loss in precision due to lightweight modifications. The experimental results showed that the average precision of the improved network for sugarcane stem node identification reached 97.6%, which was 0.6% higher than that of the YOLOv5 baseline network. Meanwhile, a model size of 2.6MB, 1,129,340 parameters, and 7.2G FLOPs, representing respective reductions of 82%, 84%, and 54.4%. Compared with mainstream one-stage target detection algorithms such as YOLOv4-tiny, YOLOv4, YOLOv5n, YOLOv6n, YOLOv6s, YOLOv7-tiny, and YOLOv7, G-YOLOv5s-SS achieved respective average precision improvements of 12.9%, 5.07%, 3.6%, 2.1%, 1.2%, 3%, and 0.4% in sugarcane stem nodes recognition. Meanwhile, the model size was compressed by 88.9%, 98.9%, 33.3%, 72%, 92.9%, 78.8% and 96.3%, respectively. Compared with similar studies, G-YOLOv5s-SS not only enhanced recognition accuracy but also considered model size, demonstrating an overall excellent performance that aligns with the requirements of sugarcane seed pre-cutting machines.

## 1. Introduction

Sugarcane, an invaluable crop of substantial economic significance, serves as the primary raw material not only for sugar production but also as a vital resource for the light, chemical, and energy sectors. Presently, over 90 countries globally engage in sugarcane cultivation, encompassing a vast planting expanse of approximately 22 million hectares. The primary sugarcane-producing countries are Brazil, India, China, Thailand, the United States, and Australia [1]. Nonetheless, traditional methods of sugarcane cultivation grapple with several challenges: they are labor-intensive, time-consuming, and marked by inefficiency, considerably constraining the progress of the sugarcane industry [2]. Consequently, there is an imperative need for the advancement of mechanized and intelligent practices within the sugarcane cultivation domain. Indeed, given the imperative of segmenting sugarcane to achieve a heightened germination rate and minimize seed consumption during the cultivation process, combined with the growth of sugarcane buds on the stem nodes, it becomes paramount to preserve the integrity of these stem nodes during seed cutting. Hence, the realization of automatic, highly efficient, and precise stem node identification emerges as a pivotal technological breakthrough, poised to elevate the industrial production of sugarcane seeds and foster the mechanization of sugarcane cultivation.

Existing sugarcane stem node identification methods are mainly divided into traditional machine vision and deep learning methods. Traditional machine learning algorithms have matured significantly and found extensive application [3–5]. In the study of sugarcane stem nodes, conventional machine learning methods primarily employ features such as texture, grayscale, color, and edges of the sugarcane for identification. For example, Huang et al. [6] studied a sugarcane stem node identification method based on the local mean algorithm. Their method estimated the position of the sugarcane stem node by calculating the average gray value and determining the corresponding position of the maximum average gray value. The obtained identification rate was 90.77% with an average identification time of 0.48 s. Nonetheless, this approach is impacted by the selected stride and fixed template width, rendering it unable to address the entirety of a sugarcane. Shi et al. [7] introduced a machine vision-based approach aimed at tackling the challenge of identifying sugarcane internodes across diverse varieties and under varying background conditions, achieving a recognition rate of 92%. Nevertheless, the precision of this approach diminishes in scenarios involving intricate image backgrounds. Based on the characteristics of the inflection point and discontinuous gray value at the sugarcane node, Zhang et al. [8] adopted edge fitting, gray value fitting, and median decision methods to identify sugarcane nodes, with an identification rate of 94.7%. However, this method exhibits a pronounced sensitivity to fluctuations in the illumination conditions. Chen et al. [9] have presented an approach for sugarcane stem nodes recognition based on wavelet analysis, achieving a remarkable detection rate of 99.63% and a rapid response time of 0.25 seconds. Nevertheless, it fails to meet the demands of real-time detection. Chen et al. [10] proposed a sugarcane stem node recognition algorithm based on the sum of local pixels with respect to the smallest point of the vertical projection function. Their algorithm achieved an identification rate of 100% and average response time of 0.15 s for a single node, and an identification rate of 98.5% and average response time of 0.21 s for double nodes. However, this method exclusively focuses on single and double nodes and does not encompass the recognition of an entire sugarcane. This analysis highlights that conventional machine vision methods exhibit slow recognition speeds and are unable to promptly and accurately identify the entire sugarcane. Additionally, they come with demanding environmental requirements. When the background undergoes complex changes, the recognition rate, robustness, and generalization ability decrease, posing challenges for practical usage.

Recently, deep learning models represented by convolutional neural networks have developed rapidly. As very effective classification and recognition models, they have attracted considerable attention worldwide, been widely used [11–15], and achieved good results in the agricultural field. Examples include fruit identification [16, 17], crop diseases and pests identification [18, 19], animal behavior detection [20, 21], etc. Studies have creatively applied deep learning to identify sugarcane stem nodes. Li et al. [22] applied YOLOv3 to the recognition of sugarcane stem nodes. Their study improved the original YOLOv3 network by reducing the number of residual structures, changing the size of the output feature map, and reducing the number of anchors. The average precision increased by 2.26%, and the average identification time, which was 51.5 ms, reduced to 28.7 ms compared with those of the original YOLOv3. Tang [23] optimized the YOLOv4 model to improve sugarcane stem node recognition accuracy and speed. The strategies adopted include introducing an effective feature layer obtained through the backbone into the enhanced feature extraction network, reconstructing the path aggregation, and changing the Mish activation function in the original model to a Leaky ReLU activation function. Chen et al. [24] introduced the YOLOv4 algorithm to study the recognition of sugarcane stem nodes in a natural environment and realize fast and accurate recognition of sugarcane stem nodes in a complex natural environment. Wang et al. [25] proposed an algorithm aimed at enhancing YOLOv4-Tiny for sugarcane stem node recognition. This improved algorithm achieved remarkable results, including a mean accuracy precision of 99.11%, a detection accuracy of 97.07%, and a transmission frame per second (fps) rate of 30. As evident from the algorithm described above, deep learning-based target detection methods utilize intricate network architectures to extract valuable feature information from images, enabling effective target detection in challenging environments. In contrast to conventional machine learning approaches, deep learning methods offer enhanced robustness, superior generalization abilities, quicker detection speeds, and are better suited for real-time detection tasks.

However, the aforementioned research on sugarcane stem node recognition algorithms based on deep learning primarily emphasizes enhancing algorithm accuracy and detection speed, with little attention given to the model’s lightweight characteristics. A highly complex model demands greater memory and computing power, which contradicts the trend of equipment miniaturization and significantly raises the cost of mechanizing sugarcane planting for mobile and embedded devices. Therefore, this study proposes a lightweight network based on YOLOv5. Its objective is to condense the model’s size, diminish its complexity, attain high-precision detection and identification, and enhance detection efficiency to align with the needs of sugarcane production tasks.

The main contributions of this study are summarized as follows:

1. The lightweight design of YOLOv5 comprehensively considers the performance and complexity of the model and determines the better network architecture.
2. The complexity of the YOLOv5 model is further reduced by using the Ghost module to replace the conventional convolution module in the BottleNeck structure.
3. The loss of precision in the lightweight process is compensated and detection ability of the model on sugarcane stem nodes is improved by introducing the SimAM attention mechanism.
4. A performance test was conducted by comparing the proposed method with the current mainstream one-stage target detection algorithm, concluding that the G-YOLOv5s-SS proposed in this study attains a higher average precision and lower model complexity. In other words, it effectively achieves a lightweight model while maintaining high detection accuracy.

## 2 Materials and methods

### 2.1 Data acquisition

The dataset samples used in this study were taken from Fusui County, Guangxi Sugarcane Production Collaborative Innovation Center. The selected sugarcanes had a growth period of approximately 6 to 8 months, with an average diameter of 30 mm. In total, 540 sugarcane samples were selected and divided into three batches of 180. Image acquisition was performed using an intelligent transverse sugarcane seed pre-cutting machine developed by Guangxi Minzu University.

**Fig 1:**
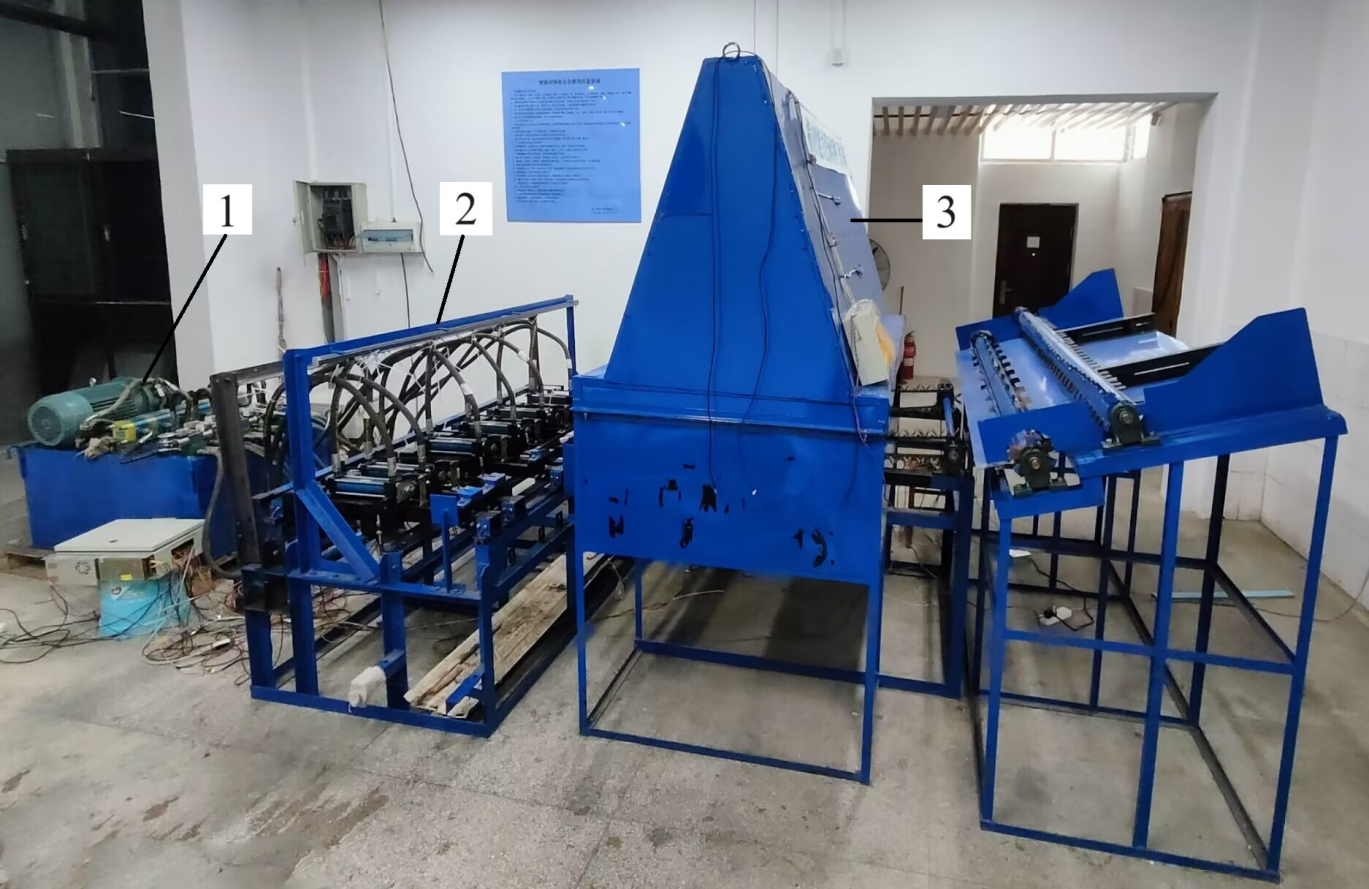
The main components of the sugarcane seed pre-cutting machine. 1. Hydraulic device 2. Seed-cutting device 3. Sugarcane node identification device.

**Fig 2:**
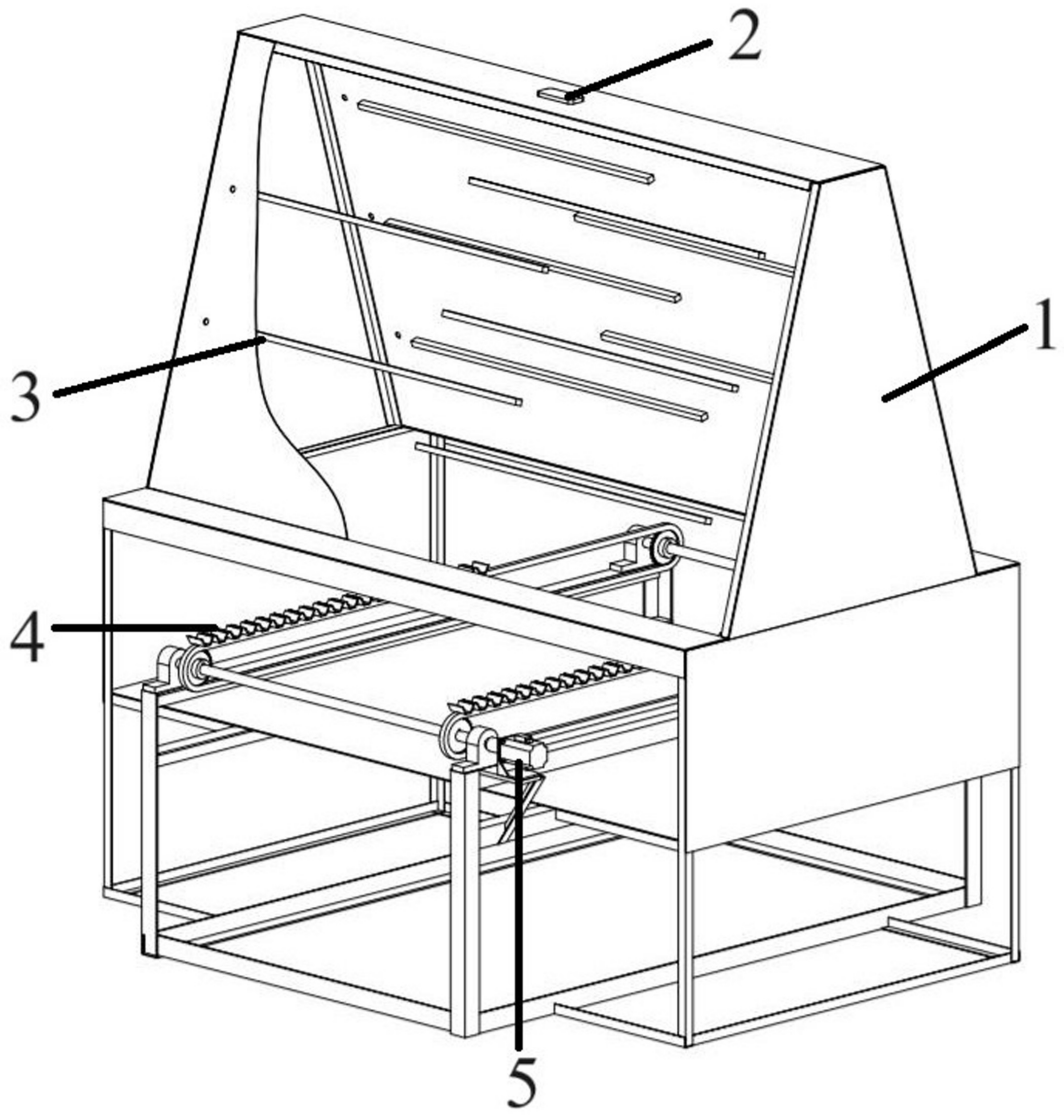
Schematic of sugarcane node identification device. 1. Black box 2. Camera 3. Strip LED light 4. Conveyor chain mechanism 5. Stepper motor.

**Fig 3:**
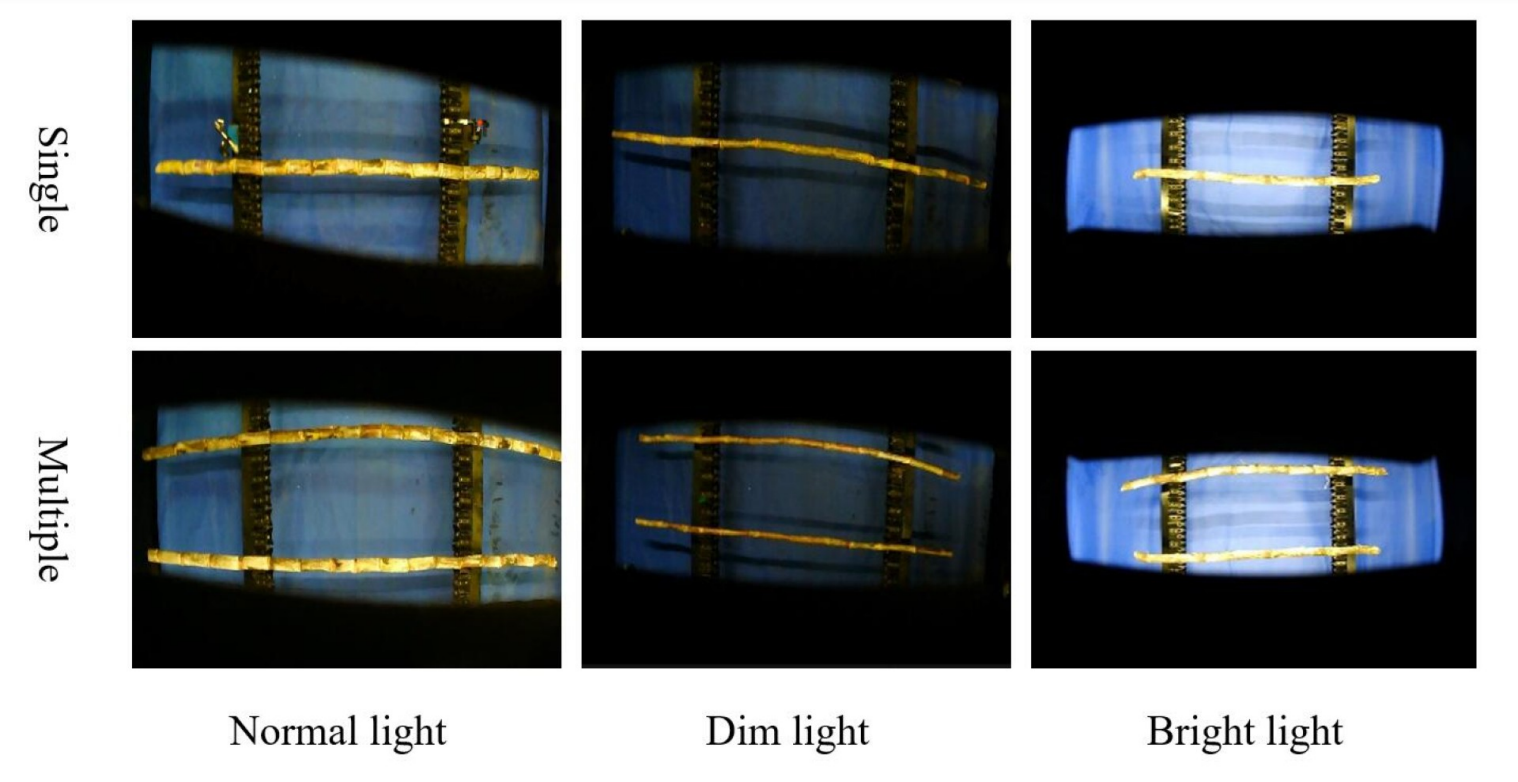
Some sample pictures.

The structure of the intelligent transverse sugarcane seed pre-cutting machine is shown in Fig 1. It is mainly composed of three parts: a hydraulic device, a seed-cutting device, and a sugarcane node identification device. The hydraulic device is the power source of the seed-cutting device. The sugarcane node identification device as shown in Fig 2, consists of a black box, a camera, ten strip LED lights, a conveyor chain mechanism, a stepper motor, and a data processing computer. The conveyor chain mechanism is powered by a 57-step stepper motor. An industrial camera is installed 1.5 m above the conveyor chain mechanism. Its model is RMONCAM-G200 (1080P), with a resolution of 1920 × 1080 and a video frame rate of 20 frames/s. Ten strips of LED lights are uniformly distributed on both sides of the black box. Each strip of LED light is 1 m long emitting white light. The sugarcanes were horizontally transmitted through the camera field of view by the conveyor chain mechanism for video capture, and the data samples were obtained by taking screenshots from the captured videos. To enrich the diversity of data and improve the generalization ability of the model, sugarcane images were collected under different lighting conditions and in different numbers, as shown in Fig 3. The lighting conditions were set as normal light, dim light, and bright light; the sugarcane number emerging at the camera field of view was set as single and multiple. Finally, 870 images, including approximately 16,000 stem nodes, were collected and divided into sets of 700 training, 70 verification, and 100 test images.

### 2.2 Improvement of the YOLOv5

**Fig 4:**
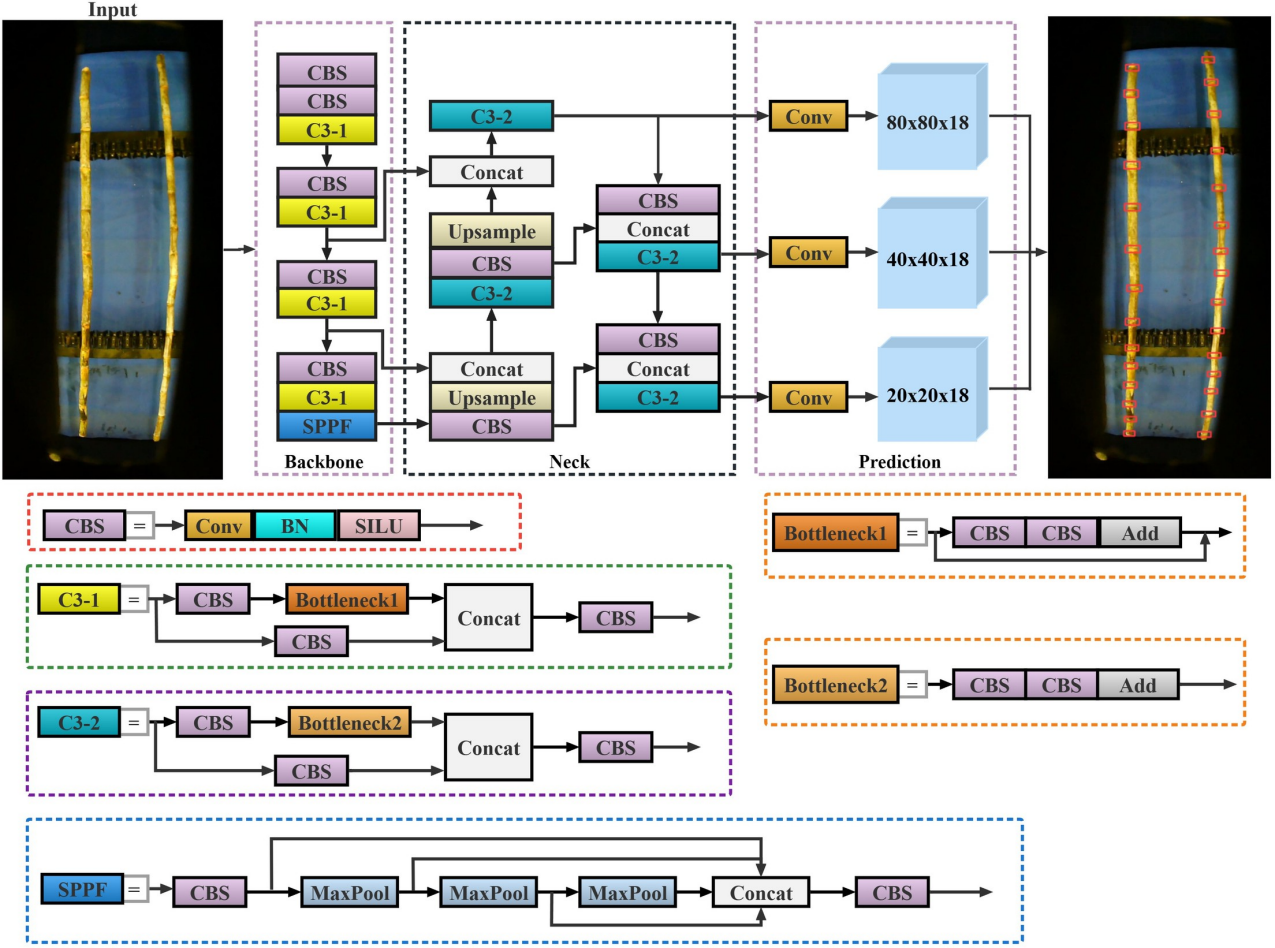
Network structure of YOLOv5.

YOLOv5 is an improved one-stage target-detection algorithm based on YOLOv3 [26]. Compared with other algorithms in the field of target detection, YOLOv5 has the characteristics of a small model size, fast training and reasoning speed, and flexible use; thus, has been widely used in various fields [27–29]. Fig 4 shows the network structure of YOLOv5. To enable YOLOv5 to identify sugarcane stem nodes more quickly and accurately and meet the actual use requirements of the intelligent transverse sugarcane seed pre-cutting machine, the following improvements are proposed in this study.

#### 2.2.1 Lightweight improvement of the network structure

**Fig 5:**
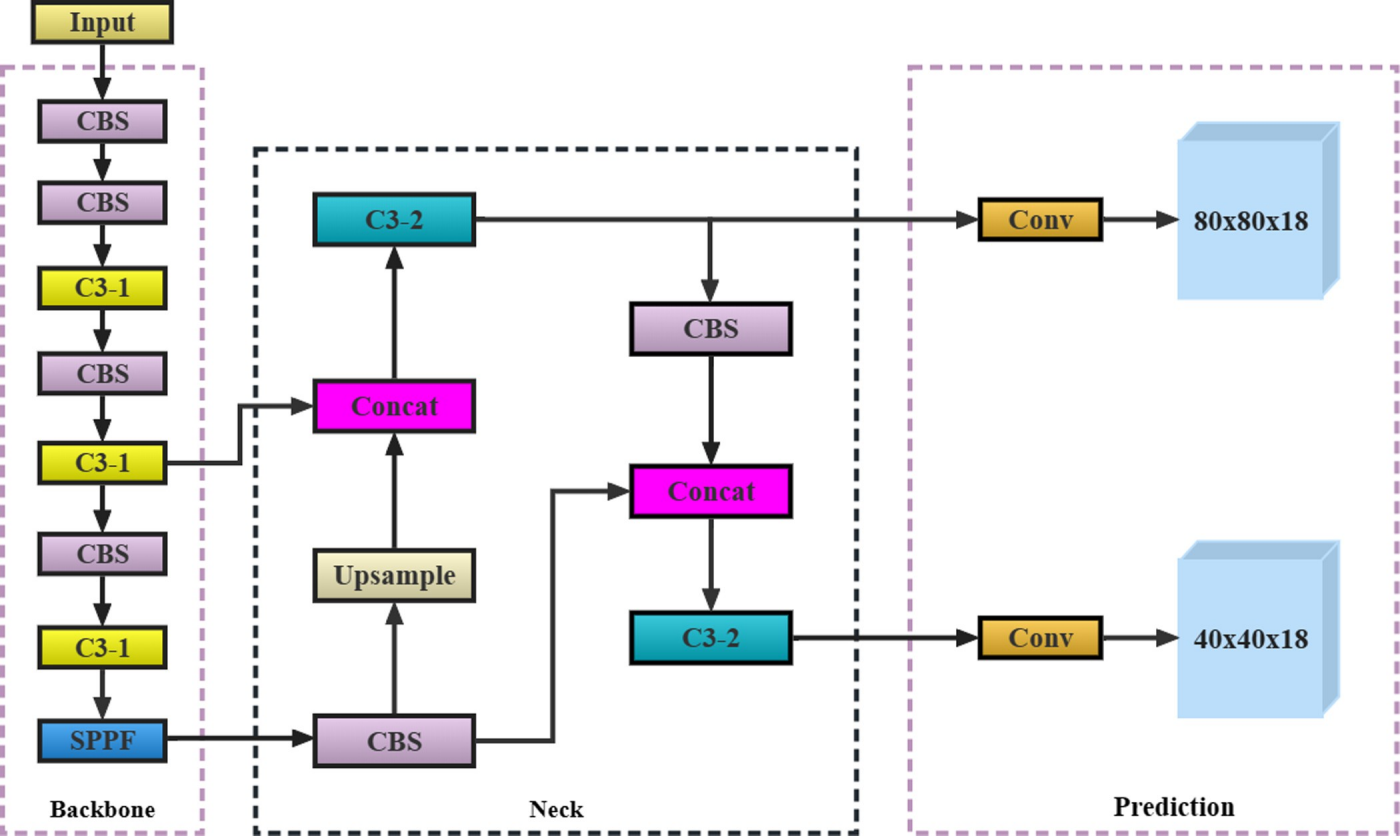
YOLOv5 lightweight structure.

From the data acquisition process described in Section 2.1, we establish that the sugarcane nodes occupy only a small pixel area in an image. However, the YOLOv5 network structure considers all small-, medium-, and large-target detections. During feature extraction, the feature information of small targets mostly exists in a shallow network. To obtain richer semantic information and improve the detection ability of large targets, the backbone network of YOLOv5 requires a deeper network structure to extract deep features. As the network deepens, the number of output feature channels exponentially increases. Therefore, most of the parameters of the entire network structure are concentrated in the last few layers of the backbone network. Moreover, in the neck structure, YOLOv5 adopts 32, 16, and 8 down-sampling times, generating 20 × 20, 40 × 40, and 80 × 80 feature maps to detect the large, medium, and small objects, respectively. In other words, YOLOv5 is designed redundantly to detect sugarcane nodes. To ensure the lightweight design of YOLOv5, both the sugarcane node size and working principle of an intelligent transverse sugarcane seed pre-cutting machine should be considered. Additionally, the horizontal distance between the industrial camera and conveyor chain mechanism is fixed, and the pixel size of the sugarcane nodes does not change significantly in an image. Thus, the branch of the 20 × 20 detection scale played a small or even negligible role in the identification of sugarcane nodes. Subsequently, the following strategies for lightweight improvements on YOLOv5 are proposed: first, the P7 and P8 layers are removed from the backbone network, changing the down-sampling rate from 32 to 16 times. Second, 32 times down-sampled branches were removed from the neck structure, and the 20 × 20 network structure was deleted from the FPN and PAN structures. Compared with the original network, the lightweight network reduces the number of down-samplings and adopts a smaller receptive field. It can reduce the model parameters and amount of computation while retaining more fine-grained feature information, thus, improving the detection effect of sugarcane stem nodes. The improved network structure is shown in Fig 5.

#### 2.2.2 Ghost-C3 module

To further reduce the number of parameters and calculations, this study introduces the so-called Ghost module [30] into the network. The original YOLOv5 network used regular convolution modules to generate numerous of rich and redundant feature maps. Although these redundant feature maps ensure the detection accuracy and generalization ability of the model, they also significantly increase the number of parameters and computation complexity. Contrarily, the Ghost convolution uses a simple linear transformation to generate phantom features to replace the redundant features generated by regular convolution. It obtains numerous effective feature maps with fewer parameters and computations, uses a small amount of regular convolutions to calculate the feature map, and processes these feature maps through cheap linear transformation to generate numerous phantom features. Finally, the two sets of feature maps are spliced using identity mapping to form a new output.

**Fig 6:**
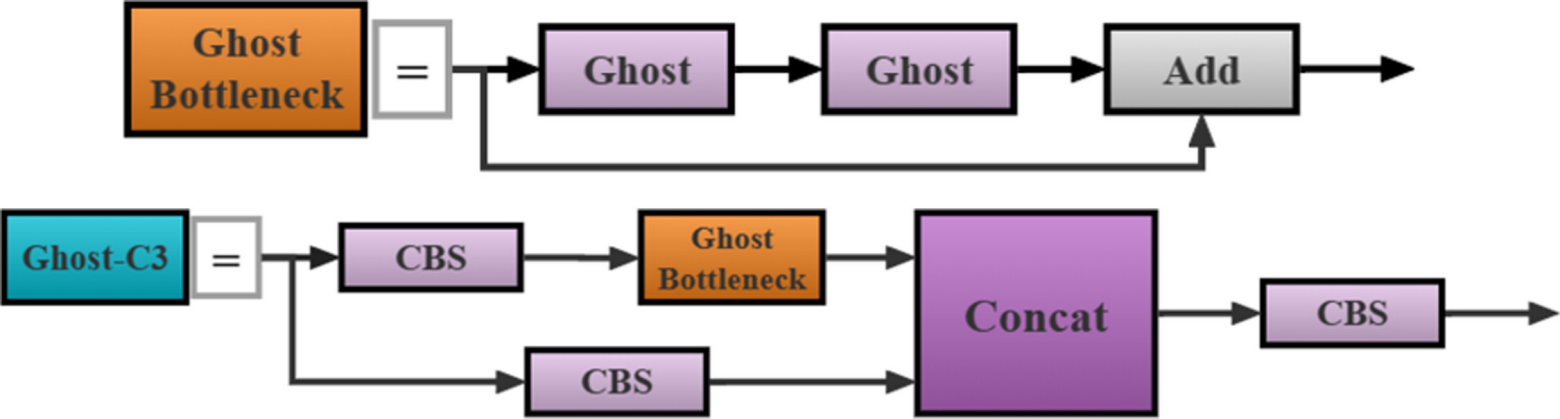
Construction of the Ghost-C3 Lightweight module. a. Ghost-BottleNeck structure, b. Ghost-C3 structure.

Based on the above analysis, this study designed the so-called Ghost-C3 structure. The structure adopts the Ghost convolution to replace the regular convolution to form a Ghost-BottleNeck structure, as shown in Fig 6A. Then, the Ghost-BottleNeck is used to replace the BottleNeck module in the original YOLOv5 network to form the Ghost-C3 structure, as shown in Fig 6B. Finally, the improved Ghost-C3 module is used to replace all C3 modules in the backbone and neck networks. The introduction of the Ghost-C3 module further reduces the number of parameters and computation complexity without reducing the number of features that affect the detection accuracy of the model. Accordingly, it significantly improves the reasoning speed of the model, and thus, improves the working efficiency of the intelligent transverse sugarcane seed pre-cutting machine.

#### 2.2.3 The SimAM attention module

A lightweight model structure often results in a loss of detection accuracy and reduces the performance of the model. To improve the detection accuracy regarding sugarcane nodes, this study introduces the SimAM attention mechanism [31] to the detection model. The SimAM attention mechanism is based on the visual neuroscience theory. Neurons with rich neuroscience information usually exhibit different discharge patterns from peripheral neurons and inhibit peripheral neurons. Neurons with spatial inhibitory effects on visual processing should be allocated higher importance. In the task of detecting sugarcane stem nodes, the neurons of these networks often need to be enhanced to improve the extraction efficiency of sugarcane stem node features and improve detection accuracy. These neurons can be detected by measuring the linear separation between a target neuron and other neurons. Based on these results, the SimAM attention mechanism yields the following energy function:

$$e_{t}^{*}=\frac{4\left({\hat{\sigma }}^{2}+\lambda \right)}{{\left(t-\hat{\mu }\right)}^{2}+2{\hat{\sigma }}^{2}+2\lambda }$$ (1)

$$\hat{\mu }=\frac{1}{M}\sum_{i=1}^{M}x_{i}$$ (2)

$${\hat{\sigma }}^{2}=\frac{1}{M}\sum_{i-1}^{M}{\left(x_{i}-\hat{\mu }\right)}^{2}$$ (3)

where t denotes the target neuron and x denotes the peripheral neuron.

In Eq (1), the lower the energy $e_{t}^{*}$, the greater is the difference between neuron t and peripheral neuron x, and the higher the importance of this neuron. Therefore, the importance of each neuron can be obtained by $1/e_{t}^{*}$. The final SimAM module is optimized based on the following:

$$\tilde{X}=sigmoid\left(\frac{1}{E}\right)⊙X$$ (4)

where X is a neuron in a single channel of the input feature, $\tilde{X}\;$ is a neuron in a single channel of the output feature, E is used to classify the energy function $e_{t}^{*}$ in all channels and spatial dimensions, and the sigmoid function is used to limit the possible excessive values in E.

Moreover, in contrast to the existing 1D channel attention and 2D spatial attention mechanisms, the SimAM attention mechanism can derive 3D attention weights for the feature map without adding additional parameters, enabling the network to learn more discriminative neurons and improve the feature extraction ability. This is a simple but highly effective attention mechanism module.

## 3. Experimental design and results analysis

### 3.1 Experimental environment and parameter setting

The processing platform used in this study is a notebook computer equipped with the operating system Windows 11, a 3.20 GHz AMD Ryzen 7 5800H CPU, 16 GB of memory, and an NVIDIA GeForce RTX 3060 (6GB) GPU. To improve the speed of network training, GPU acceleration was utilized. The CUDA version was 11.6.0, and the cuDNN version was 8.3.2. The development language was Python 3.8.13, and the PyTorch 1.12.1, with an initial learning rate of 0.01, a final OneCycleLR learning rate of 0.01, a momentum of 0.937, weight decay of 0.0005, a batch size of 8, and 200 epochs. Each model was trained using a transfer learning method.

### 3.2 Model evaluation indexes

This study aims to design a lightweight YOLOv5 model while maintaining a high detection accuracy. Therefore, it is necessary to evaluate the performance and complexity of the model. Commonly employed evaluation metrics encompass average precision (AP), model size, number of parameters, and floating-point operations (FLOPs). The AP value is the area of the P-R curve composed of the accuracy rate P and recall rate R, as expressed in Eqs 5–7.


$$\text{AP}=\int_{0}^{1}\text{P}(\text{R})\text{dR}\;\times \;100\text{\%}$$ (5)

$$\text{P}=\frac{\text{TP}}{\text{TP}+\text{FP}}\;\times \;100\text{\%}$$ (6)

$$\text{R}=\frac{\text{TP}}{\text{TP}+\text{FN}}\;\times \;100\text{\%}$$ (7)

Here, TP denotes the correct detection number of sugarcane stem nodes. FP denotes the incorrect detection number of sugarcane stem nodes, where negative samples are depicted as positive samples, and FN denotes the incorrect detection number of sugarcane stem nodes, where positive samples are depicted as negative samples.

The model size corresponds to the storage space required by the model, which is mainly determined by the number of parameters in the model. The smaller the model is, the easier it is to apply to mobile terminals. FLOPs denote the number of floating-point operations, which are used to measure the calculation amount and complexity of the model. Generally, the lower the FLOPs value is, the less inference time is required by the model.

### 3.3 Selection of the benchmark model

Based on the network depth and width, there are five different YOLOv5 network models, namely, YOLOv5n, YOLOv5s, YOLOv5m, YOLOv5l, and YOLOv5x. From the YOLOv5 official documentation, the performances of these models are improving but their complexity increases correspondingly. Owing to the large difference between the self-built sugarcane stem node dataset and COCO dataset, the data comparison among the models given by the YOLOv5 official documentation was not sufficient to obtain satisfactory results when selecting the benchmark model. Therefore, the performances of the sixth version of the YOLOv5 network model were tested on the self-built dataset, as shown in Table 1. The AP values of YOLOv5s, YOLOv5m, YOLOv5l, and YOLOv5x were almost the same, all of which were approximately 97%. However, in terms of model size, number of parameters, and FLOPs, the latter three are more than 2 times that of YOLOv5s, among which YOLOv5x has the highest complexity, and 12 times, 12.3 times, and 12.9 times that of YOLOv5s, respectively. We can see that the complex network structure does not improve the detection accuracy of sugarcane stem nodes but increases the model size and calculation burden. Although YOLOv5n is superior to YOLOv5s in model size, parameters, and FLOPs, its AP value is only 94%, lower than 97% of YOLOv5s. Moreover, its network depth and width are not enough for subsequent improvement. After comprehensive consideration, this study chooses YOLOv5s version 6.0, which possesses relatively balanced performance and complexity, as the benchmark model.

**Table 1:** Detection results of YOLOv5 series models on the sugarcane stem node dataset.

**Model**	**AP (%)**	**Model size (MB)**	**Parameters**	**FLOPs (G)**
YOLOv5n	94	3.9	1760518	4.1
YOLOv5s	97	14.4	7012822	15.8
YOLOv5m	97	42.2	20852934	47.9
YOLOv5l	96.9	92.2	46108278	107.6
YOLOv5x	97	173.1	86173414	203.8

### 3.4 Lightweight experiment of the YOLOv5s baseline network structure

Based on the analysis presented in Section 2.2.1, the YOLOv5s baseline network structure can be designed in a lightweight manner by removing certain modules. Accordingly, we deleted the convolution layer and C3 module at the end of the backbone network, and canceled the 20 × 20 detection layer, as described in Section 2.2.1. To obtain the optimal lightweight model, the following five experimental schemes are proposed: Scheme 1 uses the benchmark model YOLOv5s purely. Scheme 2 removes the P7 and P8 layers from the baseline network structure and adopts the detection scales of 160 × 160, 80 × 80, and 40 × 40. Scheme 3 removes the P7 and P8 layers from the baseline network structure and adopts detection scales of 160 × 160 and 80 × 80, respectively. Scheme 4 removes the P7 and P8 layers from the baseline network structure and adopts detection scales of 80 × 80 and 40 × 40, respectively. Scheme 5 removes layers P5, P6, P7, and P8 from the baseline network structure and adopts detection scales of 160 × 160 and 80 × 80. The experimental results are listed in Table 2.

**Table 2:** Comparison of the different lightweight schemes.

**Scheme**	**AP (%)**	**Size (MB)**	**Parameters**	**FLOPs(G)**	**network layers**
1	97	14.4	7012822	15.8	213
2	97.2	4.4	1701526	12.9	178
3	96.9	3.6	1253636	11.7	155
4	96.8	3.4	1558340	10.1	132
5	94.7	1.8	424516	8.4	111

As indicated in Table 2, Scheme 2 achieves an AP score of 97.2%, marking a 0.2% enhancement compared to the baseline model. Importantly, Scheme 2 surpasses all five other schemes in terms of AP score performance. This improvement can be attributed to the introduction of a larger 160x160 scale detection head in Scheme 2. This component effectively leverages shallow-level feature information, thereby enhancing the accuracy of sugarcane stem node detection. Regarding model size, parameters, FLOPs, and network layers, Scheme 2 exhibits superior performance compared to the baseline model but falls short of Schemes 3, 4, and 5. Scheme 3 and Scheme 4 achieve AP scores of 96.9% and 96.8%, respectively, representing a 0.1% and 0.2% reduction compared to the baseline model. However, in terms of model size, parameters, FLOPs, and network layers, Scheme 3 and Scheme 4 are only equivalent to 25%, 17.9%, 74%, 72.7%, and 23.6%, 22.2%, 63.9%, 61.9% of the baseline model, respectively. Overall, Schemes 3 and 4 exhibit superior performance to the baseline model, with Scheme 4 performing slightly better than Scheme 3. Scheme 5, on the other hand, achieves the lowest model complexity and the best lightweight effect. Nevertheless, its AP score is the lowest among the five schemes, marking a 2.3% reduction compared to the baseline model. This can be attributed to the substantial removal of modules from the backbone network in Scheme 5, which results in inadequate feature extraction for sugarcane node detection, ultimately leading to decreased accuracy. Considering both detection accuracy and lightweight effects, Scheme 4 is selected as the structure for the lightweight network and is named YOLOv5s-S.

### 3.5 Comparative experiment on the attention mechanism

To verify the effectiveness of the SimAM attention mechanism in terms of detection accuracy and lightweight effects of YOLOv5s-S, performance tests were conducted and other attention mechanism modules were introduced to conduct horizontal comparisons. The comparison attention mechanisms include SE [32], CBAM [33], ECA [34] and CA [35]. They were added to the entire network at the same location as the YOLOv5s-S backbone network, that is, after the SPPF module. The results are presented in Table 3.

**Table 3:** Comparison between different attention mechanisms.

**Model**	**AP (%)**	**Size (MB)**	**Parameters**	**FLOPs(G)**	**Network layers**
YOLOv5s-S	96.8	3.4	1558340	10.1	132
YOLOv5s-S-SE	97.2	3.5	1566532	10.1	138
YOLOv5s-S-CBAM	97.2	3.5	1566903	10.2	144
YOLOv5s-S-ECA	97	3.4	1558343	10.1	136
YOLOv5s-S-CA	97.1	3.5	1565020	10.2	142
YOLOv5s-S-SimAM	97.7	3.4	1558340	10.1	134

**Fig 7:**
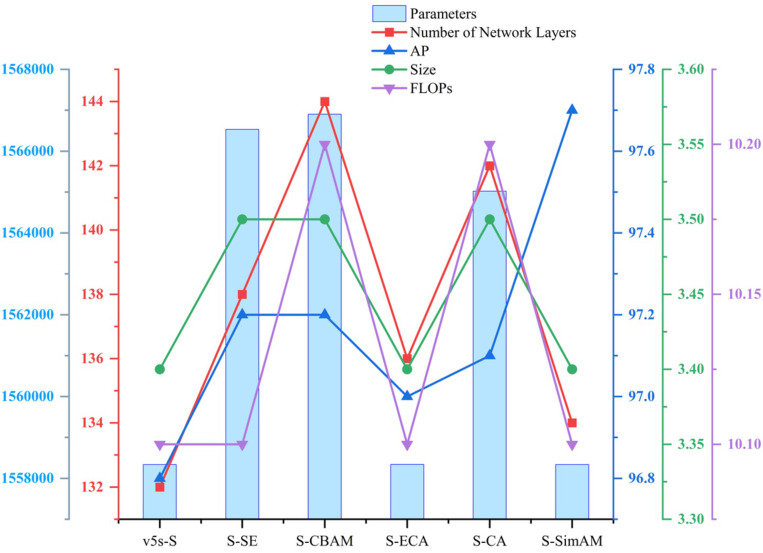
Comparison of results obtained with different attention mechanisms.

From Table 3, it is evident that introducing attention mechanisms into the YOLOv5s-S model can enhance detection accuracy to varying degrees. Among them, YOLOv5s-S-SimAM achieves the highest detection accuracy, reaching 97.7%, which is a 0.9% improvement over YOLOv5s-S. Additionally, it outperforms models incorporating SE, CBAM, ECA, and CA attention mechanisms by 0.5%, 0.5%, 0.7%, and 0.6%, respectively. In terms of complexity, YOLOv5s-S-SimAM does not introduce any additional parameters or computational burden; the model size remains unchanged, with only a two-layer increase in network layers, making its impact on model complexity negligible. In contrast, both YOLOv5s-S-CBAM and YOLOv5s-S-CA exhibit increases in model size, parameters, FLOPs, and network layers. YOLOv5s-S-SE shows increases in all metrics except FLOPs. YOLOv5s-S-ECA, while nearly unchanged in model complexity compared to YOLOv5s-S, achieves the lowest AP score at 97%, with only a modest 0.2% improvement. A visualization chart of various attention mechanism comparison results is shown in Fig 7.

From the Fig 7, it can be seen that among the columns representing parameters, v5s-S, S-ECA, and S-SimAM have similar heights, all lower than S-SE, S-CBAM, and S-CA, indicating that they have fewer parameters; In terms of model size, network layers, and FLOPs, the S-SimAM model is comparable to the v5s-S model, indicating that introducing SimAM attention does not increase model complexity. In terms of AP indicators, the S-SimAM model is higher than other models, indicating its best performance. For the self-built sugarcane dataset, the improved YOLOv5s-S-SimAM model proposed in this section strengthens the weight of the sugarcane stem node features, ensuring that the model pays more attention to the target features to be detected. While enhancing the ability to extract sugarcane stem node features, irrelevant features, such as impurities on the sugarcane surface and the background plane of the conveyor chain, are effectively suppressed. Moreover, the introduction does not add any parameters to the model. This is beneficial to the lightweight design characteristic of the network. Therefore, compared to other attention mechanisms, adding SimAM to the improved model has the best effect.

### 3.6 Ablation experiment

To verify the importance of the lightweight network structure, Ghost-C3 module, and SimAM attention mechanism module for sugarcane stem node detection, YOLOv5s was selected as the baseline network for the ablation experiment. Three networks and a baseline network are proposed. No. 1 is a lightweight structure designed using the baseline network. No. 2 uses Ghost-C3 modules to replace all C3 modules in the baseline network. Section 3 introduces the SimAM attention mechanism to the baseline network. The experimental results are listed in Table 4.

**Table 4:** Results of ablation experiments.

**Model**	**Lightweight**	**Ghost-C3**	**SimAM**	**AP(%)**	**Size(MB)**	**Parameters**	**FLOPs(G)**
YOLOv5s				97	14.4	7012822	15.8
1	√			96.8	3.4	1558340	10.1
2	√	√		96.6	2.6	1129340	7.2
3	√	√	√	97.6	2.6	1129340	7.2

**Fig 8:**
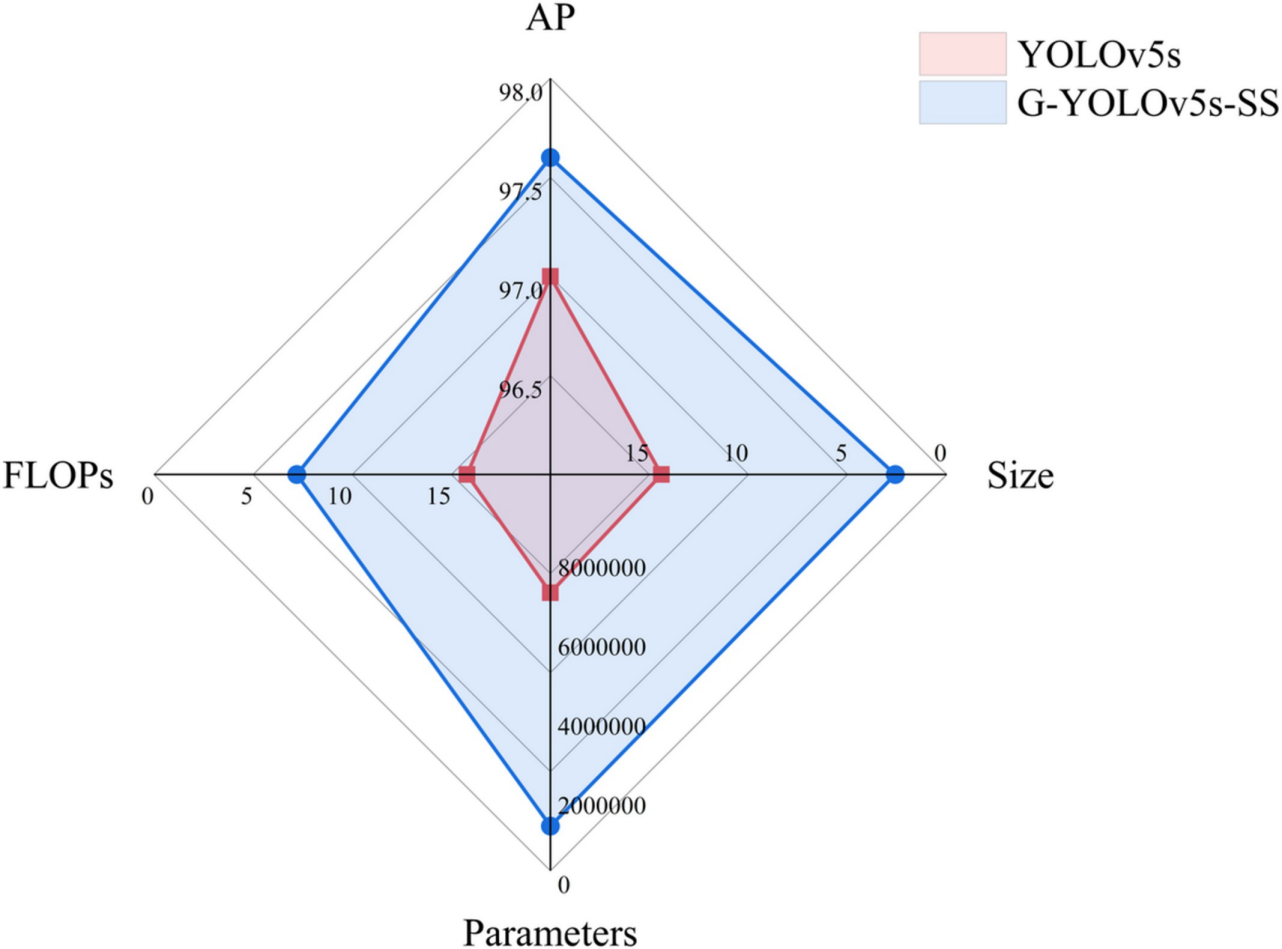
Comparison before and after model improvement.

After the lightweight improvement of the baseline network, the complexity of the model was significantly reduced compared with that of the original network. For the No.1 scheme, the number of parameters is reduced by 77.8%, model size is compressed from 14.4MB to 3.4MB, FLOPs is only 63.9% of that of YOLOv5s, and average precision is only reduced by 0.2%. After replacing the original C3 module with Ghost-C3 module in Scheme 1, the model size, number of parameters, and FLOPs of Scheme 2 are reduced by 23.5%, 37%, and 28.7%, respectively, compared with those of Scheme 1, further reducing the model complexity with an average precision that is reduced by only 0.2%. After the introduction of the SimAM attention mechanism, Scheme 3 achieves an average precision that is 1% higher than that of Scheme 2, thereby compensating for the accuracy loss caused by Scheme 1 and Scheme 2, while maintaining other indicators unchanged. The final improved model is named G-YOLOv5S-SS. A comparison of the G-YOLOv5S-SS and YOLOv5s baseline models is shown in Fig 8.

In the radar chart (Fig 8) above, the red area represents the baseline model YOLOv5s, while the blue area corresponds to the model proposed in this study, G-YOLOv5s-SS. As depicted in the chart, G-YOLOv5s-SS exhibits significant reductions in model size, parameters, and FLOPs compared to YOLOv5s. Furthermore, the AP value exhibits a slight improvement. The results of the ablation experiments demonstrate that the proposed model excels in terms of performance and complexity when compared to YOLOv5s, thereby meeting the identification requirements for sugarcane nodes in seed-cutting machines.

### 3.7 Comparison experiment of the mainstream one-stage algorithms

To further verify that the improved G-YOLOv5s-SS network is superior to the other algorithms, comparative experiments were performed between the G-YOLOv5s-SS and mainstream one-stage algorithms. The mainstream one-stage algorithms considered were YOLOv4 [36], YOLO5, YOLOv6 [37], and YOLOv7 [38]. The experiment was conducted under the same conditions; the results are presented in Table 5.

**Table 5:** Detection results of different single-stage target detection algorithms.

**Model**	**AP (%)**	**Size (MB)**	**Parameters (M)**	**FLOPs (G)**
YOLOv4-tiny	84.7	23.6	5.87	16.2
YOLOv4	92.53	244.2	63.93	141.9
YOLOv5n	94	3.9	1.76	4.1
YOLOv5s	97	14.4	7.01	15.8
YOLOv6n	95.5	9.29	4.3	11.06
YOLOv6s	96.4	36.7	17.18	44.07
YOLOv7-tiny	94.6	12.3	6.01	13
YOLOv7	97.2	71.3	36.5	103.2
G-YOLOv5s-SS	97.6	2.6	1.13	7.2

**Fig 9:**
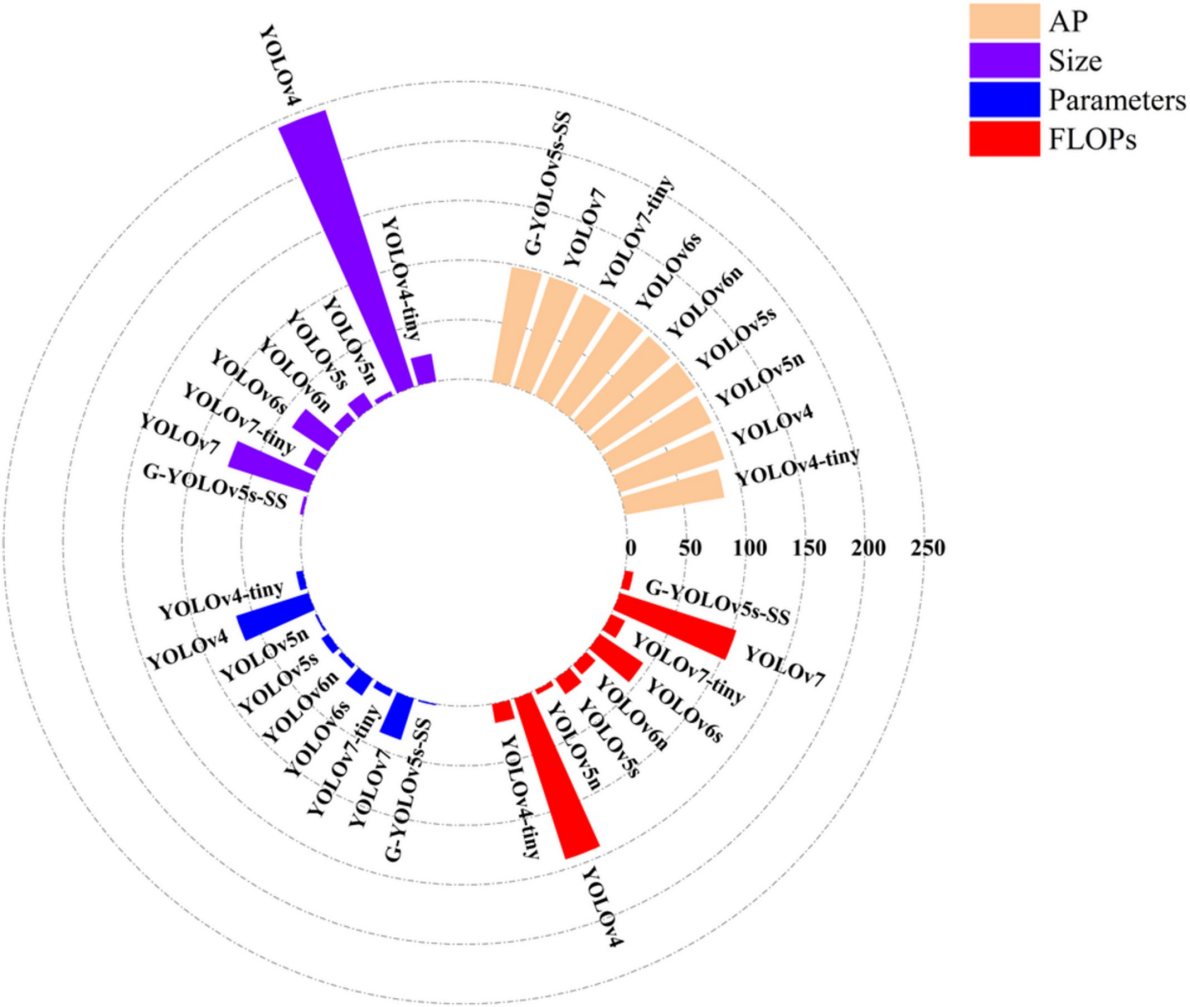
Comparison of different one-stage detection algorithms.

We can see from Table 5 that on the self-built sugarcane dataset, the G-YOLOv5s-SS proposed in this study has higher detection accuracy compared with the single-stage target detection algorithms, namely, YOLOv4, YOLOv5s, YOLOv6s, and YOLOv7. Meanwhile, the model size is compressed by 98.9%, 82%,92.9% and 96.3%, respectively; the parameters and the FLOPs are reduced by 98.2%,84%,93.4%,96.9% and 94.9%, 54.4%, 83.6%, 93%, respectively. Compared with other lightweight networks, such as YOLOv4-tiny, YOLOv5n, YOLOv6n, and YOLOv7-tiny, the AP values of the G-YOLOv5s-SS model is increased by 12.9%, 3.6%, 2.1%, and 3%, respectively. In terms of complexity, except for FLOPs, which are slightly larger than that of YOLOv5n, the model performed well. Fig 9 provides a comparison of the visualization results.

From the Fig 9, it is evident that the AP of G-YOLOv5s-SS is slightly higher compared to other models. However, when evaluating metrics such as model size, parameters, and FLoPs, G-YOLOv5s-SS exhibits lower numerical values than the other models. Therefore, this model is better suited for enhancing the accuracy and efficiency of sugarcane seed pre-cutting machines.

## 4. Discussion

### 4.1 Visualization analysis

**Fig 10:**
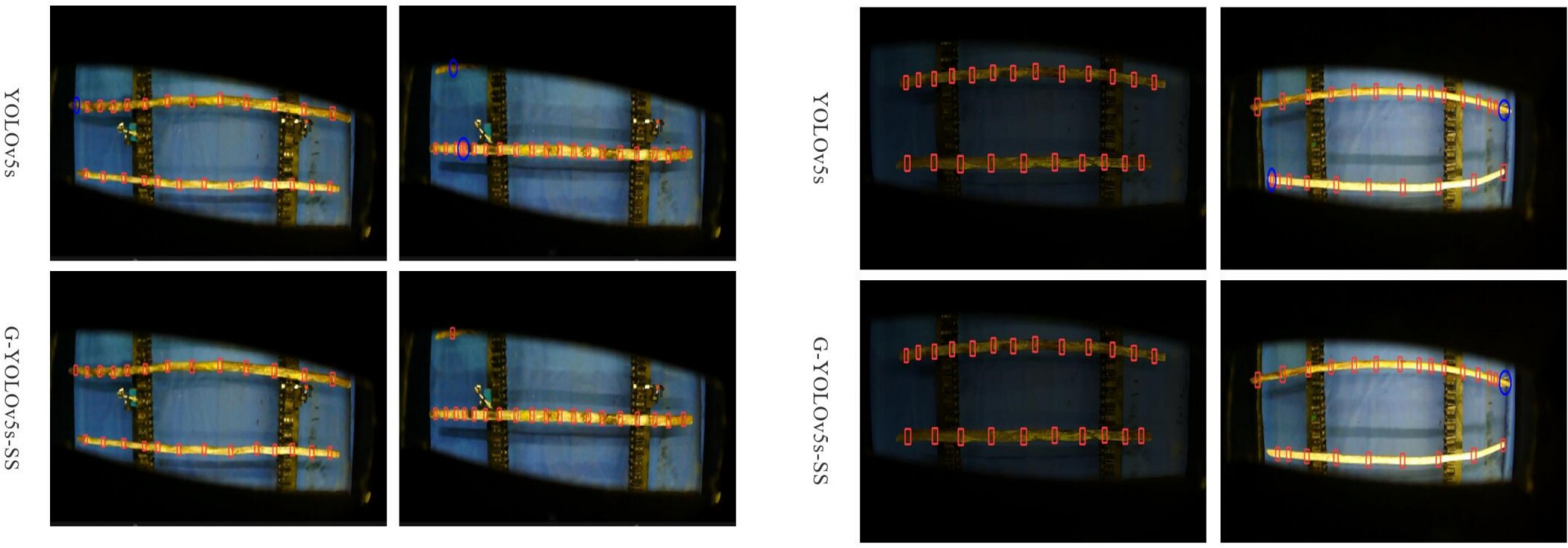
Visualization of detection results. a. Detection effect under normal light conditions. b. Detection effect under dim and strong light conditions.

From Section 3.7, the improved network G-YOLOv5s-SS is superior to the baseline network in terms of performance. Particularly, the AP value of G-YOLOv5-SS reaches 97.6%, 0.6% higher than YOLOv5s. To compare the detection effects of the two algorithms more intuitively, parts of the visualization results are shown in Fig 10 on the test set. In the test image, the red box denotes the location of the stem node marked by the model, while the blue oval circle denotes the location where the model has a false or missed inspection and is marked manually. Fig 10A shows the detection of sugarcane nodes under normal light conditions. In the first column, G-YOLOv5s-SS can completely identify all sugarcane stem nodes but YOLOv5s misses one node at the left end. In the second column, YOLOv5s labels the negative samples as positive samples and misses one node at the upper left corner, while G-YOLOv5s-SS can accurately identify the sugarcane stem nodes. Fig 10B shows the detection of sugarcane stem nodes under dim (first column) and strong light conditions (second column). In the first column, both algorithms can completely and accurately identify the sugarcane nodes. In the second column, the two algorithms fail to recognize the three sugarcane stem nodes when the nodes are too close to each other; moreover, YOLOv5s appears to label the negative samples as positive samples.

Therefore, G-YOLOv5s-SS has a better actual detection effect. To ensure the detection accuracy of G-YOLOv5s-SS, the lighting conditions in the sugarcane seed pre-cutting machine should be adjusted appropriately. In this case, the advantages of G-YOLOv5s SS over the other algorithms can be fully demonstrated. Moreover, a high-resolution industrial camera can improve the quality of the self-built sugarcane dataset. The distance between sugarcanes and speed of the conveyor chain mechanism used in the sugarcane node identification device should also be considered.

### 4.2 Comparative analysis of different methods

In a similar research on sugarcane stem node recognition, Li et al. [22] utilized deep learning techniques to achieve earlier detection of sugarcane stem nodes. They enhanced the original YOLOv3 network by reducing the number of residual structures, modifying the output feature map dimensions, and decreasing the number of anchors. This optimization resulted in an average precision of 90.38% with an identification time of 29ms. Compared to conventional computer vision methods, their approach enabled the recognition of entire sugarcane stems and significantly improved recognition speed, meeting the requirements of real-time detection. Nevertheless, the early YOLOv3 model still had scope for accuracy improvement and remained relatively bulky in size. Chen [39] adopted two methods to improve YOLOv5s in a lightweight manner: one is to use the lightweight network MobileNet to improve YOLOv5s, and the other is to use a network-thinning algorithm to prune the sugarcane stem node recognition model based on YOLOv5s. The average precision of the two improved methods was 92.1%, which was 0.8% lower than the average precision of the benchmark model. In terms of model size, parameters, and FLOPs, the first method achieves a reduction to 65% (8.97MB), 64% (4539542), and 60% (9.8G) of those of the benchmark model, while the second method achieves a reduction to 28% (3.89MB), 14%961352), and 57% (9.3G) of those of the benchmark model. Indeed, this scholarly work has established the foundation for a comprehensive dataset containing images of sugarcane stem nodes captured under field conditions. This study effectively resolves the issue of sugarcane stem node recognition in natural environments, all while achieving model lightweighting. Nonetheless, it is noteworthy that the lightweight model, despite its efficiency advantages, does exhibit a marginally diminished detection accuracy compared to the benchmark model. Zhao et al. [40] improved YOLOv5 by enhancing information fusion capabilities, refining the loss function, and introducing the Ghost module. These enhancements led to an impressive average precision of 97.8%. Additionally, they reduced the model’s size to 78% (11.4MB) and its parameters to 80% (5.67M) of the original model. The approach presented in this literature resulted in a notable 1.4% increase in AP compared to the benchmark model while also accounting for the challenging background clutter that may occur during the operation of the seed cutting machine. It is important to mention that the changes in model size and parameters were relatively modest, with a slight increase in recognition time. In this study, G-YOLOv5s-SS achieved an AP of 97.6%, surpassing the baseline model by 0.6%. Additionally, the model’s size, parameters, and FLOPs were reduced to 18% (2.6MB), 16% (1,129,340), and 45.6% (7.2G) of the baseline model, respectively. Compared to the literature mentioned earlier, the G-YOLOv5s-SS proposed in this study comprehensively considered both model performance and complexity. It not only enhanced detection accuracy but also significantly reduced the model’s parameter count and computational complexity. The comparison of the results obtained in this study and those reported in the literature [22, 39, 40] are reported in Table 6.

**Table 6:** Comparison of sugarcane stem node identification methods.

**Methods**	**AP (%)**	**Size (MB)**	**Parameters**	**FLOPs (G)**	**Time (ms)**
Li et al. [22]	90.38	×	×	×	29
Chen wen [39]	92.1	8.97	4539542	9.8	4.4
Chen wen [39]	92.1	3.89	961352	9.3	4.2
Zhao et al. [40]	97.8	11.4	5.67M	×	16.9
G-YOLOv5s-SS	97.6	2.6	1129340	7.2	4.3

### 4.3 limitations

However, this study also has its limitations. Firstly, in the investigation of sugarcane stem node recognition, the lighting conditions of sugarcane seed pre-cutting machines can affect the recognition accuracy of the model. Through visual analysis, it can be seen that the brightness of the LED lights on both sides of the black box has a certain impact on the experimental results, especially when the light is too bright, the recognition effect of the model on sugarcane stem nodes will degrade. Hence, for optimal model recognition results in real-world applications, it is imperative to fine-tune the lighting conditions by adjusting the light source accordingly. Furthermore, in sugarcane stem node recognition experiments on sugarcane seed pre-cutting machines, the process requires the complete removal of sugarcane leaves to improve the model’s recognition accuracy. However, Mechanized elimination of sugarcane leaves may result in residue. In actual seed cutting operations, the comprehensive elimination of sugarcane leaves requires a significant allocation of labor and resources, yielding suboptimal efficiency. Conversely, the preservation of some sugarcane leaves negatively impacts the model’s recognition performance. Therefore, future research will consider identifying sugarcane stem nodes without fully peeling off sugarcane leaves to improve the model’s recognition effect in complex environments and enhance the efficiency of the sugarcane seed pre-cutting machines.

## 5. Conclusion

To address the issues of low recognition efficiency and high demands on device memory and computational capacity in sugarcane seed cutting machines, this study presents a sugarcane stem node recognition method called G-YOLOv5s-SS, based on the improved YOLOv5s framework. Firstly, a lightweight network structure is obtained by reducing the number of downsampling times and detection layers in the backbone network. On this basis, the Ghost module is introduced to further reduce the complexity of the model. Finally, the SimAM attention mechanism is added to compensate for accuracy loss. The accuracy of G-YOLOv5s SS reached 97.6%, which is 0.6% higher than YOLOv5s. The model size, parameters, and FLOPs are 2.6MB, 1,129,340, and 7.2G, respectively, which are equivalent to 18%, 16%, and 45.6% of YOLOv5s. The following conclusions were obtained through the experiments described in this study:

1. By reducing the number of downsampling times and detection layers in the backbone network, the complexity of the model can be significantly reduced. This method can reduce the evaluation indicators such as model size, parameters, and FLOPs to 23.6%, 22.2%, and 63.9% of the benchmark model, while only reducing the average accuracy by 0.2%.
2. By introducing the Ghost module, the complexity of the model can be further reduced. After incorporating the Ghost module into a lightweight network structure, the model size was compressed to 2.6MB, and the parameters and FLOPs were reduced to 1,129,340 and 7.2G, respectively. At the same time, the AP was reduced by only 0.4% compared to YOLOv5s.
3. By adding the SimAM attention mechanism, detection accuracy can be improved. After introducing the SimAM attention mechanism, the AP reached 97.6%, an increase of 0.6% compared to the original YOLOv5s, compensating for the accuracy loss caused by lightweight improvements. Meanwhile, the model size, parameters, and FLOPs remain unchanged, which does not affect the complexity of the model.
4. Compared with mainstream one-stage object detection algorithms (YOLOv4 tiny, YOLOv4, YOLOv5n, YOLOv6n, YOLOv6s, YOLOv7 tiny, and YOLOv7), the AP values of G-YOLOv5s SS have been improved by 12.9%, 5.07%, 3.6%, 2.1%, 1.2%, 3%, and 0.4, respectively, while the model size has been compressed by 88.9%, 98.9%, 33.3%, 72%, 92.9%, 78.8%, and 96.3%, respectively. Compared with similar studies, G-YOLOv5s SS balances detection accuracy and complexity, and performs well overall.

The experiment proves that the G-YOLOv5s-SS proposed in this study has high detection accuracy and low complexity, which can meet the working requirements of sugarcane seed pre-cutting machines. In future research, we will consider identifying sugarcane stem nodes in complex environments to further improve the efficiency of sugarcane seed pre-cutting machines.
