# Correction: Sugarcane stem node identification algorithm based on improved YOLOv5

**DOI:** 10.1371/journal.pone.0298247

**Published:** 2024-01-31

**Authors:** Zhongjian Xie, Yuanhang Li, Yao Xiao, Yinzhou Diao, Hengyu Liao, Yaya Zhang, Xinwei Chen, Weilin Wu, Chunming Wen, Shangping Li

The order of the affiliations is incorrect. Please view the correct affiliation order here:

Zhongjian Xie ^1,3^, Yuanhang Li ^1,3^, Yao Xiao ^1,3^, Yinzhou Diao ^1,3^, Hengyu Liao ^1,3^, Yaya Zhang ^1,3^, Xinwei Chen ^1,3^, Weilin Wu ^1,2^, Chunming Wen ^1,3^, Shangping Li ^1,3^

**1** College of Electronic Information, Guangxi Minzu University, Nanning, China, **2** Guangxi Key Laboratory of Machine Vision and Intelligent Control, Wuzhou University, Wuzhou, China, **3** Guangxi Key Laboratory of Hybrid Computation and IC Design Analysis, Guangxi Minzu University, Nanning, China
